# Does mental rotation emulate motor processes? An electrophysiological study of objects and body parts

**DOI:** 10.3389/fnhum.2022.983137

**Published:** 2022-10-11

**Authors:** Marta Menéndez Granda, Giannina Rita Iannotti, Alexandra Darqué, Radek Ptak

**Affiliations:** ^1^Laboratory of Cognitive Neurorehabilitation, Department of Clinical Neurosciences, Faculty of Medicine, University of Geneva, Geneva, Switzerland; ^2^Swiss Foundation for Innovation and Training in Surgery, University Hospitals of Geneva, Geneva, Switzerland; ^3^Division of Neurorehabilitation, University Hospitals of Geneva, Geneva, Switzerland

**Keywords:** mental rotation, ERP, embodiment, simulation, angular gyrus, parietal lobe

## Abstract

Several arguments suggest that motor planning may share embodied neural mechanisms with mental rotation (MR). However, it is not well established whether this overlap occurs regardless of the type of stimulus that is manipulated, in particular manipulable or non-manipulable objects and body parts. We here used high-density electroencephalography (EEG) to examine the cognitive similarity between MR of objects that do not afford specific hand actions (chairs) and bodily stimuli (hands). Participants had identical response options for both types of stimuli, and they gave responses orally in order to prevent possible interference with motor imagery. MR of hands and chairs generated very similar behavioral responses, time-courses and neural sources of evoked-response potentials (ERPs). ERP segmentation analysis revealed distinct time windows during which differential effects of stimulus type and angular disparity were observed. An early period (90–160 ms) differentiated only between stimulus types, and was associated with occipito-temporal activity. A later period (290–330 ms) revealed strong effects of angular disparity, associated with electrical sources in the right angular gyrus and primary motor/somatosensory cortex. These data suggest that spatial transformation processes and motor planning are recruited simultaneously, supporting the involvement of motor emulation processes in MR.

## Introduction

The evolution and use of higher cognitive functions are submitted to strong biological constraints, which provide an upper bound on complexity, interdependence, and energy consumption. Increasing evidence shows that the human brain deals with these constraints by reutilizing basic motor and sensory resources for higher cognitive processing (Wilson, 2002; Anderson, 2010; Dehaene et al., 2010). While an intense debate exists regarding the degree to which symbolic knowledge is embodied (Hauk et al., 2004; Caramazza et al., 2014), even critics agree that some form of embodiment must underlie mental rotation (MR; Goldinger et al., 2016). Proponents of embodiment propose that cognitive processes have their origins in bodily functions or representations (Thelen, 2000; Barsalou, 2008; Anderson, 2014). They are critical of an information processing approach that considers decontextualized stimulus-response relationships which are based on symbolic representations (such as advocated by Fodor and Pylyshyn, 1988). Instead, they propose that representations in the brain are modal, depend on the context and are in the service of action. Regarding MR, the embodied approach is based on several arguments. First, a well-established linear relationship exists between reaction time and degree of the rotation stimulus, expressed as angular disparity from a zero-degree (upright) reference item (Shepard and Metzler, 1971; Searle and Hamm, 2017). This pattern is what would be expected from a process that emulates the temporal characteristics of a physical rotation, for example when subjects create a mental image and then manipulate it until a match with the probe is found. Second, MR capacity seems to run parallel to motor development, as motor control (assessed as the ability to collect and manipulate matches or sticks) is an independent predictor of MR performance in young children (Jansen and Heil, 2010). Third, functional neuroimaging studies show that MR activates regions of posterior parietal and premotor cortex similar to those involved in motor planning, suggesting strongly overlapping neural substrates (Filimon, 2010; Vingerhoets, 2014; Ptak et al., 2017; Cona and Scarpazza, 2019). We refer to motor planning as those processes that enable the collection and integration of information on actions in order to concretize an intended movement, i.e., processes which are necessary before a movement can be executed (Hoshi and Tanji, 2007; Wong et al., 2015).

Though these observations suggest that MR and motor planning may share embodied neural mechanisms, the degree of overlap between cognitive processes involved in MR of objects and body parts is not well established (Tomasino and Gremese, 2015). Based on thorough task analyses several cognitive stages have been described that appear to be consistently involved in MR: a perceptual stage, which encompasses stimulus discrimination and identification of its current orientation, a rotational stage and a decision stage (Heil and Rolke, 2002; Munzert et al., 2009; Boonstra et al., 2012). However, different cognitive subprocesses may be involved depending on the stimulus type that is manipulated. For instance, participants performing MR of objects often describe a visual imagery strategy: they create an image of the stimulus and then mentally rotate it until it matches the original (Jola and Mast, 2005). In contrast, MR of body parts appears to require some form of motor imagery (de Lange et al., 2006; Coslett et al., 2010). For example, when participants are asked to judge the laterality of a shown hand, they mentally simulate moving their own hand until it reaches the orientation of the hand pictured (Sekiyama, 1982; Parsons, 1987; Perruchoud et al., 2016). This egocentric process is what an account based on embodiment would predict (Amorim et al., 2006). Functional imaging studies comparing MR of objects and body parts support the involvement of distinct, yet partially overlapping cognitive processes by showing similar neural activations. Several MR studies of objects revealed bilateral activations of posterior parietal cortex (PPC), while MR of hands more often activated motor and premotor areas (Cohen et al., 1996; Kosslyn et al., 1998; Richter et al., 2000; Jordan et al., 2002; Wraga et al., 2005; Lamm et al., 2007; Milivojevic et al., 2009). Meta-analyses support the involvement of posterior parietal and motor/premotor regions in MR, but they do not distinguish between different types of stimuli (Zacks, 2008; Ptak et al., 2017; Cona and Scarpazza, 2019). A more recent meta-analysis examined differences between activations related to MR of bodily and non-bodily stimuli and found that the former activated frontoparietal areas including lateral premotor and supplementary motor cortices while the latter generated right superior and inferior parietal activations (Tomasino and Gremese, 2015). However, few of the included studies compared the two types of stimuli directly and with the same subjects. Vingerhoets et al. (2002) compared MR of tools and body parts and found premotor activations when subjects performed MR of hands and tools, although the former showed greater activity in right precentral cortex. While there is some evidence that MR of objects and body parts both rely on similar neural substrates and motor emulation processes, at least three important limitations remain. First, manipulable objects (such as tools) might activate motor areas simply because they are automatically associated with a specific hand action. Second, activations of motor/premotor areas to object rotation might at least partly be linked to the fact that subjects answered with a finger press. Finally, even if fMRI studies show common activations for objects and body parts, they cannot resolve the temporal order of brain regions activated in quick succession.

We here took advantage of the excellent temporal resolution of the EEG. Modern high-resolution EEG allows identification of electrical current sources with improved precision, thus providing an acceptable trade-off for the study of physiological processes varying in space and time. Previous ERP studies seem to agree on the distinction of two relevant time windows: an early phase (∼170 to 200 ms) that might be associated with visual classification or identification of the visual stimulus, followed by a later phase reflecting the mental transformation of the image in the mind (∼400 to 700 ms; Pegna et al., 1997; Petit et al., 2006). In this later phase, several studies have observed more negative amplitudes of ERPs over the parietal cortex as a function of angular disparity (Peronnet and Farah, 1989; Thayer and Johnson, 2006; Riecansky and Jagla, 2008; Jansen et al., 2020). Petit et al. (2006) even observed a right parietal activation to inverted pictures of a manipulable (hammer) and a non-manipulable object (church). However, there is a lack of ERP studies comparing MR of objects and body parts directly. The closest to answering our question whether MR of all types of stimuli relies on motor emulation comes a recent study by Jansen et al. (2020). These authors compared MR of geometric figures, whole human bodies and body postures, and identified an early period ∼200 to 400 ms after stimulus onset, reflecting distinct activation patterns between abstract and embodied figures over parietal and central electrodes. In addition, a late period (∼400 to 600 ms) was characterized by discrete ERP patterns over central and frontal electrodes. However, this study emphasized differences, rather than pointing to similarities between MR of bodily stimuli and objects. For this reason, further comparisons are necessary to determine the precise degree of overlap between cognitive processes and underlying neural sources of activations. In sum, while previous work strongly points to shared processes and shared underlying anatomical structures for MR of hands and tools, this evidence is distributed across studies. In our point of view, an ideal study that would strongly support a motor emulation component should show that MR processes for hands and stimuli that do not afford specific hand actions are closely similar regarding timing and share a common anatomical substrate. It is also important to narrow down the concept of motor emulation. We have proposed to refer to a subcomponent of motor planning that stores the abstract kinematics of an action (e.g., in the case of rotation a “turn-left” or “turn-right” operation), without assigning it to a specific effector (Ptak et al., 2021). While previous studies suggest the presence of embodied processes in MR, it is difficult to assign these processes to motor emulation as defined above.

The aim of the present study was to examine to what extent MR of objects that do not afford a specific hand action (such as grasping or shaking) and bodily stimuli (hands) shares cognitive processes, time courses and neural sources. ERPs are sensitive to stimulus characteristics, mental operations and decision processes, and we attempted to create experimental conditions that differed only with regard to the stimulus type yet were comparable for all other aspects. First, participants should not use their hands to respond since this might contaminate bodily processes involved in the MR task (e.g., imagining a rotation with one’s own hand might be disrupted by answering with the same hand; Wohlschlager and Wohlschlager, 1998; Wexler et al., 1998); for this reason, we asked participants to respond verbally while maintaining always the same body posture. Second, the response options should be exactly the same (i.e., to respond by saying “left” or “right”), irrespective of the type of stimulus. Third, since we suspected greater hemispheric differences in MR between a verbal and non-verbal stimulus than between two non-verbal items, different to other studies (Krause et al., 2021) we did not consider stimuli with a strong link to verbal functions (such as letters). We also wanted the task to be object-based for both types of stimuli and, if possible, to rely on egocentric processes, which are recruited in left-right judgment (Amorim et al., 2006; Voyer et al., 2017). Finally, the object should not be automatically associated with a specific action requiring hand movements, such as tools. In order to comply with all these conditions, we chose chairs as object stimuli. Though chairs can be touched, moved, pushed, they do not afford a specific hand action and are suitable for left-right discrimination judgments. We hypothesized that MR of chairs and hands (a) generate comparable behavioral and electrophysiological response patterns (i.e., increase of RT with increasing angular disparity, similar time-course of activity) and (b) both rely on neural structures involving cortical regions that are involved in motor planning (premotor cortex and posterior parietal cortex).

## Materials and methods

### Participants

Twenty-six right-handed healthy participants with normal or corrected-to-normal vision, recruited through advertisements published at the faculties of medicine and psychology of the University of Geneva, participated to this study. No participant had a history of neurological or psychiatric disorders. We excluded three participants due to technical problems. Since the power of within-subject EEG designs is highly dependent on the number of repetitions in each condition (Boudewyn et al., 2018), and we intended to obtain high quality EEG data, we excluded further five subjects from the analysis due to high levels of noise. Data from the remaining 18 participants (11 females) aged 19–39 years (M = 25.33, SD = 5.24) was used for analysis. The study was approved by the Ethical Commission of the canton of Geneva, and all research was performed in accordance with relevant guidelines/regulations. Participants gave written informed consent before being enrolled.

### Stimuli and procedure

Hands and chairs were used as rotation stimuli. MR with hands is hypothesized to rely on a MI strategy and therefore is commonly used to assess MI ability (Boonstra et al., 2012). Since this task requires participants to identify a hand shown in an upright or rotated position, we chose chairs for the control condition because they can easily be seen as oriented to the left or right when shown in their canonical (upright) position. This ensured that participants produced the same kind of response for both types of stimuli (saying “left” for the left hand/leftward oriented chair, and “right” for the right hand/rightward oriented chair). Stimuli were black and white photographs of three right hands (two feminine, one masculine) seen palm-down, and three different types of chairs. For each stimulus type a mirror image was produced and for each left/right stimulus a rotated version at six different angular disparities (0°, 60°, 120°, 180°, 240°, and 300°) was created. Stimuli were 21.4° along their longer axis (16.1° along their short axis) and were shown in gray-scale on black background.

Participants were tested in a sound-proof EEG chamber. Stimuli were presented centrally on a computer screen located 57 cm in front of the participants using E-prime (E-studio) software, while EEG recordings were continuously registered. Cocksworth and Punt (2013) have shown that manual responses may interfere with MI in a hand rotation task. For this reason, we registered oral responses instead of manual RTs. Participant’s hands were placed on their knees and covered with a cloth to prevent subjects from looking at their hands while performing the task. The oral response was recorded with a microphone placed about 10 cm in front of the participants’ lips.

Before running the experiment, participants practiced 25 trials to get familiarized with the task. Each trial began with the presentation of a central fixation cross for 1,000 ms to indicate the upcoming stimulus. Participants were asked to maintain fixation on the cross and to suppress eye blinks during the trials. The rotation stimulus was then presented for 2,000 ms, and participants gave their oral response. There were nine trials for each rotational disparity (0°, 60°, 120°, 180°, 240°, and 300°) and stimulus orientation (left/right) shown in randomized order, resulting in 108 trials per block. Hands and chairs were presented in distinct blocks, with five blocks of each stimulus type. Half of the participants started with hands, the other half with chairs. Participants were told that only the responses “right” and “left” were allowed and were instructed to answer as quickly and accurately as possible, in order to ensure a low number of false responses. The experiment lasted approximately one and a half hour. Each block was followed by a short break, and a larger break (∼5 mins) was allowed before starting the second half of the experiment.

### Behavioral analyses

Oral responses were registered individually in files of 2,000 ms starting at target onset. RTs and responses were analyzed offline with a custom-made MATLAB^®^ -script, which allowed visualizing the sound wave in a figure, listening to each sound and measuring RTs by setting a mark at sound onset with the mouse. Error rates and median RTs (correct trials only) were analyzed with a repeated-measures ANOVA, with the within-subjects factors Stimulus type (chair/hand) and Angular disparity (0°, 60°, 120°, and 180°). Where appropriate the Greenhouse–Geisser correction was applied to correct for sphericity violations. *Post hoc* Bonferroni-corrected paired *t*-tests were performed on the highest-level significant interactions.

### Electroencephalography acquisition and pre-processing

Continuous high-density electroencephalography (EEG) recordings were acquired using a 156-channel Brainvision actiCHamp system (Brain Products GmbH, Germany) at sampling frequency of 1,000 Hz. During EEG installation, electrode impedance was kept under 20 kΩ at each electrode.

Electroencephalography data pre-processing was performed in Cartool software^1^ (Brunet et al., 2011). The EEG was filtered off-line between 1 and 30 Hz. Epochs from 100 ms pre-stimulus to 600 ms post-stimulus were considered for the ERPs analysis. EEG-epochs were visually inspected to exclude epochs characterized by the presence of artifacts (eye blinks or movements, muscle contractions, sweating or environmental factors) were excluded. Artifact-free epochs were averaged per condition for each subject. Before averaging across subjects, bad channels from each participant carrying repetitive artifacts during prolonged periods were interpolated from neighboring electrodes using 3D spline interpolation (<10% interpolated electrodes) (Perrin et al., 1987).

An rANOVA with the factors Stimulus type and Angular disparity showed no statistical difference between the number of epochs accepted for each Stimulus type (*F*_1,17_ = 0.133, *p* = 0.719, η^2^ = 0.008), but revealed a significant effect of Angular disparity (*F*_2.73,46.56_ = 7.29, *p* < 0.01, η^2^ = 0.3). *Post hoc* tests showed that this difference was due to a significantly lower number of correct responses for stimuli rotated by 300° than all other disparities (all *p* < 0.01). To simplify the categorization of the data and focus on those conditions that most adequately contrasted effects of MR, we only compared EEG data of the no-rotation condition (0°) with the maximal-rotation condition (180°), as has been done in previous studies (e.g., Griksiene et al., 2019). This reduced EEG analyses to the following four experimental conditions: Chair 0°, Hand 0°, Chair 180°, and Hand 180°. We decided to reduce angular disparity to two levels for two reasons: first 0° and 180° are the conditions that maximize the involvement of MR processes. Second, while previous EEG studies focused on amplitude analyses which can be performed for several (Krause et al., 2021), ERP the clustering algorithm of segmentation analyses is highly sensitive to small differences between conditions and could identify a very complex pattern that is difficult to interpret. Also, our study focused on stimulus effects in MR, we focused on those conditions that were not (0°) or maximally (180°) affected by MR.

### Global amplitude analysis

To determine whether Stimulus type, Angular disparity and/or the interaction of both factors induced significant ERP amplitude differences across the whole electrode set, a waveform non-parametric repeated-measures ANOVA was performed as exploratory analysis with the Statistical Toolbox for Electrical Neuroimaging (STEN)^2^ (Knebel and Notter, 2012). Family-wise error (FWE) was controlled through bootstrapping (N = 1,000 permutations) for a significance level α = 0.05. In addition, we applied constraining criteria regarding electrode cluster-size (only clusters consisting of minimum seven neighboring electrodes were retained) and temporal clustering (only differences that were contiguous across at least 20 time-frames were accepted).

### Evoked-response potentials segmentation and statistical assessment

To identify periods of stable scalp voltage configurations (scalp EEG topographies or “microstates”) and capture the differences between the experimental conditions, ERP cluster segmentation was performed (Pasqual-Marqui et al., 1995; Murray et al., 2008; Brunet et al., 2011). Following visual inspection of the global field power (GFP) and dissimilarity curves of averaged ERPs for the four conditions (Chairs/Hands; 0°/180°) we restricted the segmentation to the period between 90 and 500 ms post-onset. This also allowed us to focus on electrophysiological signals that were relatively uncontaminated by processes related to the verbal response (the fastest responses being >800 ms, see results). For the segmentation, a k-means clustering algorithm with 300 randomizations was used, whereby the minimal duration of maps was set to 30 ms and cluster solutions ranging from 1 to 30 clusters were examined (Pasqual-Marqui et al., 1995; Murray et al., 2008; Brunet et al., 2011). The optimal number of topographic clusters (henceforth termed “maps”) that best described the group-averaged ERPs of the four conditions was determined with a meta-criterion which integrated the results of seven independent optimization criteria (Brechet et al., 2019).

To assess statistical differences of the obtained cluster maps between the different ERPs conditions, statistical analysis was performed using a topographic non-parametric (rank-based) randomization procedure implemented in RAGU software (Koenig et al., 2014). This approach evaluates for each map the statistical difference between conditions by permuting for a defined number of times the data of the grand averaged ERPs. The permutation-based analysis compares the presence of cluster maps to the distribution obtained under the null hypothesis. This method of controlling FWE has higher statistical power than if it were based on individual subjects’ ERPs, known to have high variance (Koenig et al., 2014). We applied 1,000 permutations and set a threshold of significance to *p* = 0.05. The difference between maps is given in terms of six parameters: first (onset) and last (offset) assignment of the ERP to the map, duration (mean of the time points assigned to a given map), area under the curve (AUC; defined as the sum of GFP during all time points assigned to the map), center of gravity (i.e., the time-point associated with the center of the GFP area) and mean GFP (the average strength of activation across all map time points) (Habermann et al., 2018).

### Electroencephalography source localization

Source localization analyses were performed in Cartool software (see footnote 1; Brunet et al., 2011). In order to estimate the neural current densities underlying EEG scalp topographies obtained with the segmentation, we computed an inverse solution matrix using standardized low resolution electromagnetic tomography (sLORETA; Pascual-Marqui, 2002), which constrains the inverse solution by minimizing the second-order spatial derivative of the current source distribution to maximize spatial coherence. We used as head model the Montreal Neurological Institute (MNI) template (including the cerebellum) and applied the Locally Spherical Model with Anatomical Constraints (LSMAC) which respects local differences of skull thickness corrects for global resistivity value (Michel and Brunet, 2019). We defined a grid consisting of 6,000 solution points distributed equally in the gray matter. Considering the obtained transformation (inverse) matrix for each subject, we then inverted in the source space (i.e., template brain) each epoch corresponding to the four experimental conditions (Chairs/Hands, 0°/180°). We then averaged for each subject the inverted epochs belonging to the same condition, and finally computed a grand average across subjects. This approach normalizes the power of the current density across all solution points and corrects for background noise, resulting in an increased Signal-to Noise ratio and improved detection of those solution points that exhibit maximal activation (Brechet et al., 2019).

Specific time windows of group-averaged ERPs in the inverse space were selected to visualize the activation of the neuronal sources based on the results of the statistic of cluster maps between conditions. To better characterize the neuronal sources in such windows, volumes representing the activation above the 95*^th^* percentile were extracted in 30 ms steps, for each condition.

## Results

**FIGURE 1:**
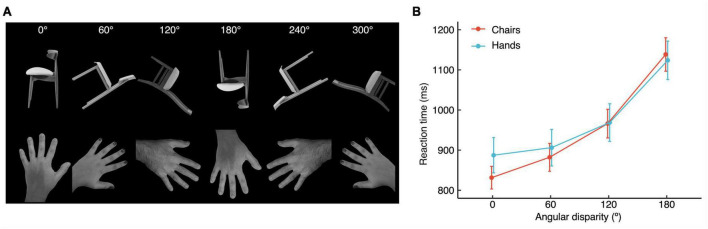
**(A)** Examples of stimulus types shown at each of the six angular disparities. **(B)** Vocal reaction times (±SEM) for hands and chairs, as a function of angular disparity.

We showed hands/chairs at different angular disparities to healthy participants and asked them to indicate whether a depicted hand represented a left or right hand, or whether a chair was oriented leftward or rightward if put in its upright position (Figure 1A). We first present the behavioral results, followed by the ERP analyses.

### Behavioral results

Figure 1B shows for both stimulus types the increase of RTs as a function of angular disparity. The rANOVA with stimulus type and Angular disparity as within-subjects factors yielded a statistically significant effect of Angular disparity on RTs (*F*_1.54,26.18_ = 106.33, *p* < 0.001, η*_*p*_*^2^ = 0.862), while Stimulus type (*F*_1,17_ = 0.281, *p* = 0.603, η*_*p*_^2^* = 0.016) was not significant, and the interaction Stimulus type × Angular disparity showed a statistical trend (*F*_1.8,30.58_ = 3.252, *p* = 0.057, η*_*p*_*^2^ = 0.161). *Post hoc t*-tests of the effect of Angular disparity revealed significant differences between all levels of disparity (all *p* ≤ 0.01). Though the interaction effect was not quite significant we also performed *post hoc* comparisons of Stimulus type at each level of angular disparity. None of these comparisons reached significance (highest *t* = 1.88_17_, *p* = 0.076).

The rANOVA also revealed a significant effect of Angular disparity on the percentage of errors (*F*_1.98,33.68_ = 4.97, *p* = 0.013, η*_*p*_*^2^ = 0.226), which was due to a higher error rate for 120° than 60° (*p* = 0.031) and 180° vs. 0° (*p* = 0.029). Stimulus type (*F*_1,17_ = 0.210, *p* = 0.652, *n*_*p*_^2^ = 0.012), and the interaction Stimulus type × Angular disparity (*F*_2,34.05_ = 0.825, *p* = 0.447, *n*_*p*_^2^ = 0.046) failed to reach significance.

### Amplitude analysis

**FIGURE 2:**
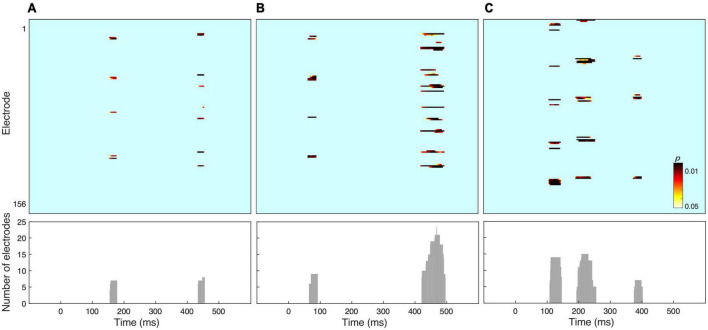
Results of electrode-wise amplitude analysis for **(A)** the stimulus effect, **(B)** the effect of angular disparity, and **(C)** an interaction between both factors. The lower panel shows the number of electrodes that reached significance at each time frame.

Figure 2 shows the results of amplitude comparisons across the whole electrode set, as a function of Stimulus type, Angular disparity or the interaction of both factors. Non-parametric waveform analyses revealed statistically significant effects of Stimulus type between ∼160 and 190 ms and Angular disparity between ∼70–100 and ∼440–500 ms. In addition, the interaction between both factors yielded significant results between ∼120–150, ∼200–260, and ∼380–410 ms. In order to examine whether these periods corresponded to EEG microstates we followed up with the ERP segmentation analysis.

### Results of evoked-response potentials segmentation

**FIGURE 3:**
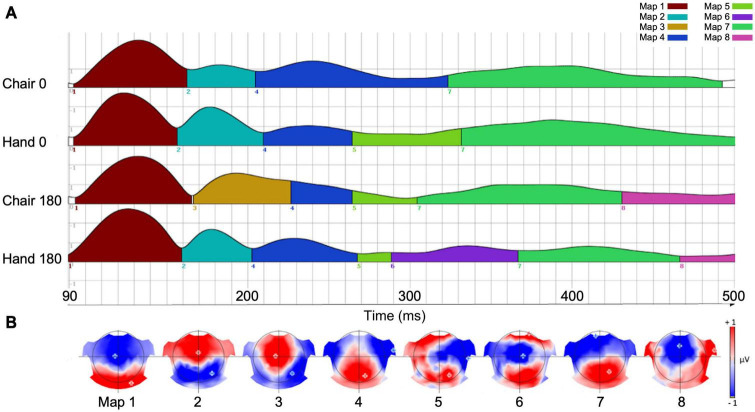
**(A)** Spatiotemporal organization of seven maps identified with the EEG segmentation procedure. The height of each curve represents Global field power at each time-point. **(B)** Current voltage topographies corresponding to each of the seven maps.

The meta-criterion for segmentation of the group-averaged ERPs associated with the four experimental conditions identified eight time-segments (Figure 3A) and associated scalp EEG topographic maps (Figure 3B). Group-averaged ERP permutation analysis across the two experimental factors (Stimulus type, Angular disparity) revealed significant results for Maps 1, 6, 7, and 8. Map 1 had an average length of 90–160 ms, corresponding to an early period of ERP differences, as reported in previous MR studies (Pegna et al., 1997). Maps 6–8 were located in a time window between 290 and 500 ms, corresponding to a late period observed in previous studies (Peronnet and Farah, 1989; Thayer and Johnson, 2006; Riecansky and Jagla, 2008; Jansen et al., 2020).

The statistical effects of each of the six map parameters are summarized in Table 1. Map 1 was only characterized by a main effect of Stimulus type on offset (*p* = 0.007; Chairs > Hands) and center of gravity (*p* = 0.006; Chairs > Hands). Map 6 was distinguished by a significant interaction for duration (*p* = 0.004) and AUC (*p* = 0.001) and was most specific for the condition Hand 180, where it was present between 290 and 360 ms. Map 7 exhibited strong main effects of Angular disparity as well as interaction effects for several parameters. It started around 310 ms for the condition Chair 180, was present for a longer period of time, ended later and had larger AUC for non-rotated than rotated stimuli (duration: *p* = 0.002; offset: *p* = 0.004; AUC *p* = 0.001). In addition, the Angular disparity effect lasted longer for Chairs than Hands (offset, interaction effect: *p* = 0.047), but had higher power for Hands than Chairs (interaction effect, AUC: *p* = 0.033; mean GFP: *p* = 0.002). Finally, it exhibited an earlier center of gravity for Chairs (interaction effect: *p* = 0.012).

**TABLE 1:** Summary of statistical effects characterizing the maps 1, 6, 7, and 8 (*p < 0.05; **p < 0.01; ***p < 0.001; AUC, area under the curve; GFP, global field power).

		Model factor		
		
	Measure	Stimulus type	Angular disparity	Interaction
**Map 1**	Onset			
	Offset	**		
	Duration			
	AUC			
	Center of gravity	**		
	Mean GFP			
**Map 6**	Onset			
	Offset			
	Duration			**
	AUC			***
	Center of gravity			
	Mean GFP			
**Map 7**	Onset			
	Offset	*	**	*
	Duration		**	
	AUC		***	*
	Center of gravity	*		*
	Mean GFP			**
**Map 8**	Onset	**	***	
	Offset			
	Duration	*	**	*
	AUC	*	***	**
	Center of gravity	**	***	
	Mean GFP			

On the grand-averaged ERPs, Map 8 (∼430–500 ms) was mainly present for rotated conditions. This map started earlier (onset: *p* = 0.0071), lasted longer (duration: *p* = 0.018) and was more activated (AUC: *p* = 0.021) for chairs than hands. In addition, it exhibited main effects of Angular disparity for onset (*p* = 0.001), duration (*p* = 0.003) and AUC (*p* = 0.001), always in favor of rotated stimuli. Angular disparity effects were longer and stronger for Chairs than Hands (interaction, duration: *p* = 0.023; AUC: *p* = 0.002).

In sum, while Map 1 indexed differences of stimulus type Map 6 was specific for rotated hands, Map 7 reflected processing of upright as compared to rotated stimuli, and this especially for chairs. In contrast, Map 8 was mainly associated with stimulus rotation, again particularly for chairs.

### Map localization

**FIGURE 4:**
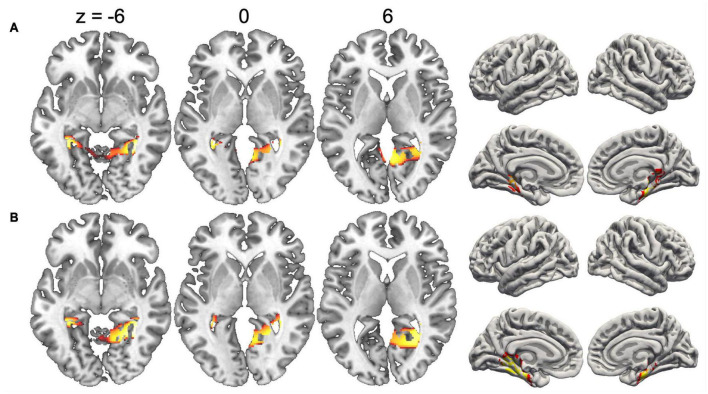
Current sources underlying Map 1 between 120 and 150 ms, projected on the MNI template brain. **(A)** Chairs and **(B)** hands. Only voxels with significance level *p* < 0.05 are shown.

**FIGURE 5:**
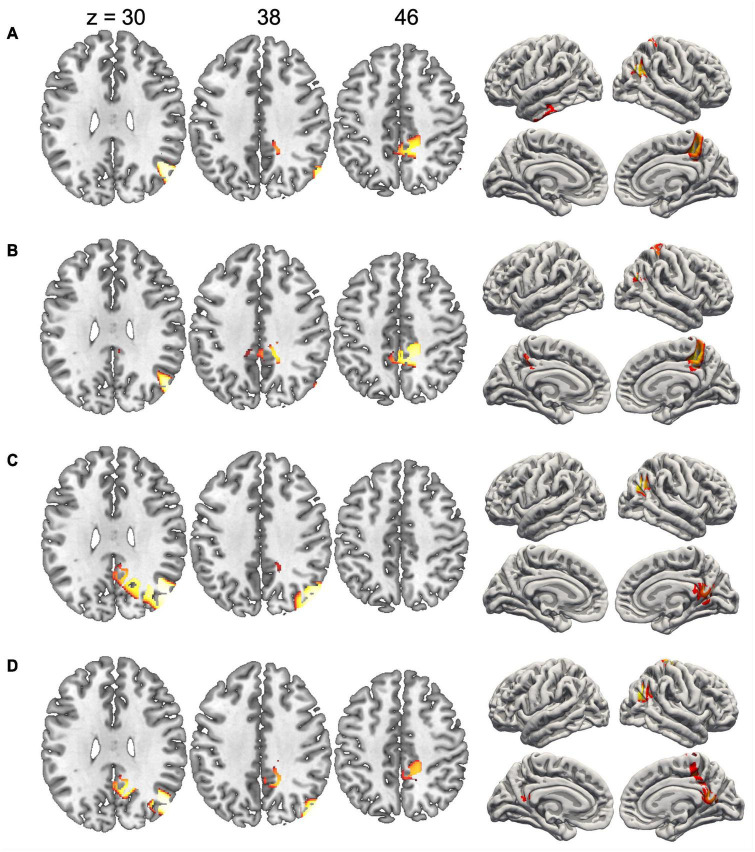
Current sources underlying Map 6 between 330 and 360 ms. **(A)** Chairs 0°, **(B)** Chairs 180°, **(C)** Hands 0°, and **(D)** Hands 180°. Only voxels with significance level *p* < 0.05 are shown.

**FIGURE 6:**
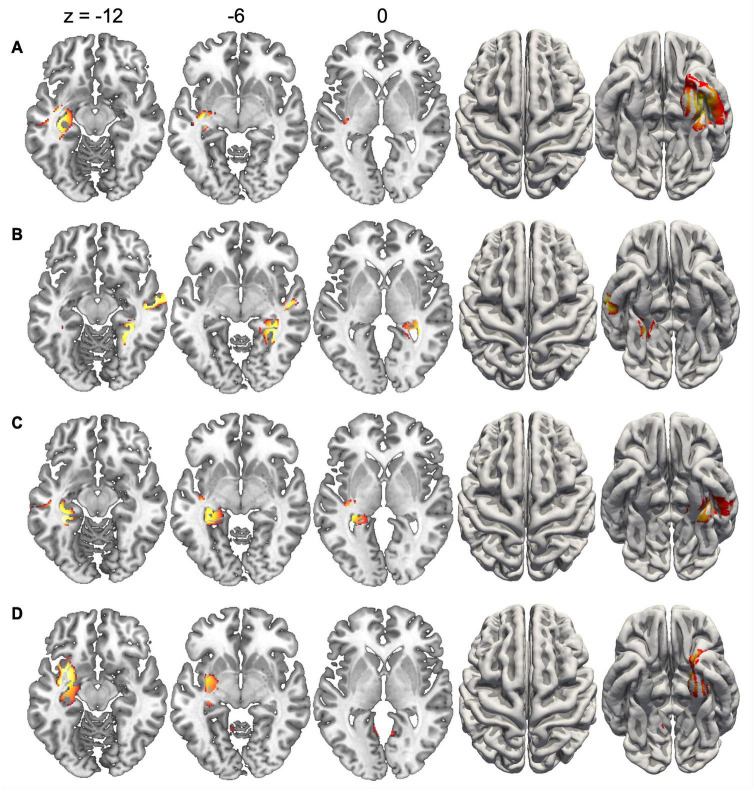
Current sources underlying Map 7 between 390 and 420 ms. **(A)** Chairs 0°, **(B)** Chairs 180°, **(C)** Hands 0°, and **(D)** Hands 180°. Only voxels with significance level *p* < 0.05 are shown.

**FIGURE 7:**
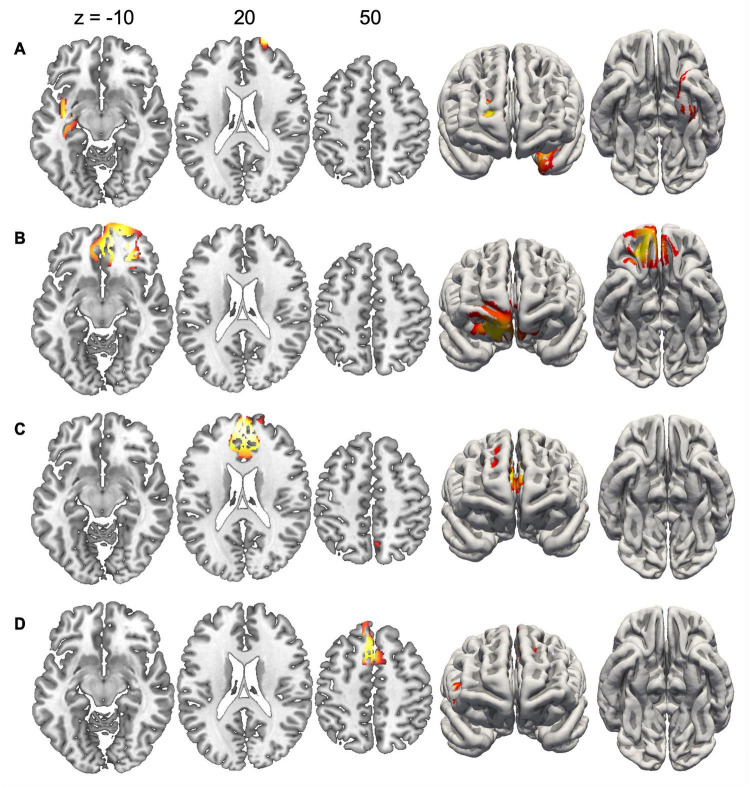
Current sources underlying Map 8 between 450 and 480 ms. **(A)** Chairs 0°, **(B)** Chairs 180°, **(C)** Hands 0°, and **(D)** Hands 180°. Only voxels with significance level *p* < 0.05 are shown.

Since the duration of maps varied across conditions, we did not compute inverse solutions across the entire map intervals, but rather over time periods when each map was particularly strongly expressed (i.e., had high GFP). Figure 4 shows the inverse solution computed over the interval 120–150 ms that was associated with Map 1 in all four conditions. Since Map 1 was only characterized by a Stimulus effect, ERP sources were computed for chairs and hands irrespective of stimulus orientation. Both stimulus types were associated with bilateral activations of fusiform and posterior parahippocampal gyrus. For hands activations reached posteriorly into the left calcarine sulcus. Current sources for Map 6 were examined in the period 330–360 ms, independently for all four conditions. This map was associated mostly with right-hemisphere activations of the angular gyrus and medial parietal cortex (Figure 5). Importantly, there was also significant recruitment of the primary somatosensory cortex and primary motor cortex. The neural current sources were strikingly different for Map 7 (390–420 ms), which was essentially characterized by left mid- and anterior temporal activations (Figure 6). An exception was the condition Chair 180, for which the inverse solution identified an area in the right medial and lateral temporal lobe. Finally, Map 8 (450–480 ms) was associated with left anterior temporal sources for Chair 0, right frontopolar sources for Chair 180 and bilateral dorsomedial sources for both hand conditions (Figure 7).

## Discussion

Our findings show that MR of hands and chairs is characterized by very similar behavioral responses, time-courses and neural sources of ERPs, suggesting a strong overlap between underlying cognitive processes. Though some previous studies also compared MR of body parts and objects (Lamm et al., 2007; Dalecki et al., 2012; Vingerhoets, 2014; Jansen et al., 2020), our MR paradigm distinguishes itself by three features. First, we used objects that are not associated with a distinctive manual response, and are therefore not expected to trigger actions implying the hands. Second, irrespective of stimulus type, subjects always had the same response options. Finally, in order to eliminate any interference of the response with motor imagery involving the hand participants were asked to respond orally. Given that the time-course of ERPs is taken as predictor of the sequence of different cognitive processes recruited during a task it is critical for EEG studies to compare conditions that are equal regarding RT and error rate. The fact that the behavioral data of hands and chairs were indistinguishable regarding RTs and accuracy makes sure that differences of ERP patterns for hands and chairs cannot be attributed to differences in processing speed.

The segmentation analysis of group-averaged ERPs for upright and rotated stimuli revealed two time periods in which stimulus type and angular disparity had differential effects. An early period (between 90 and 160 ms, corresponding to Map 1) was characterized only by an effect of stimulus type. Previous EEG studies of visual processing have revealed that high-level perceptual analysis, such as rapid recognition of faces and other body parts, as well as identification of low-level object features occur within the initial 150 ms after stimulus presentation (Johnson and Olshausen, 2003; Reed et al., 2003; Pegna et al., 2004). Given the pure effect of stimulus type characterizing early processing, Map 1 therefore seems to be related to the early rapid identification of the stimulus pictured, regardless of its orientation (which corresponds to a perceptual stage within different phases of information processing; Heil and Rolke, 2002). This interpretation is supported by the source localization analysis, showing bilateral activations in parahippocampal and fusiform gyri, brain regions that have been consistently implicated in the coding of distinct object categories (such as faces, objects or body parts) based on perceptual characteristics (Feinberg et al., 1994; Downing et al., 2006; Taylor and Downing, 2011; Ptak et al., 2014).

Effects of angular disparity and an interaction between this factor and stimulus type appeared with Map 6, and thus considerably later than the pure stimulus effect. Several previous ERP studies have observed significant differences between upright and inverted stimuli in time periods starting at ∼400 ms, an effect that has specifically been attributed to MR (Pegna et al., 1997; Jansen et al., 2020). However, while these studies suggested a single cognitive component, our segmentation and source analyses support – akin to previous modeling studies (Just and Carpenter, 1976; Xue et al., 2017) – at least three distinct processing stages. The first stage (290–360 ms) was represented by Map 6 which was mainly present when rotated hands were manipulated. The source localization revealed an activation in the right inferior parietal cortex centered on the angular gyrus, which suggests that this segment might be related to the spatial transformation of the mental images. In fMRI studies the angular gyrus is activated when subjects are engaged in visuo-spatial judgments, such as the comparison of lengths or angles (Sack et al., 2007; Sack, 2009; Singh-Curry and Husain, 2009; Seghier, 2013). It has also been consistently identified as one of the most important brain regions supporting mental image transformations in MR (Zacks, 2008; Tomasino and Gremese, 2015; Cona and Scarpazza, 2019). We found angular gyrus activation irrespective of stimulus type, and only in the right hemisphere, which is compatible with a high-level, stimulus-independent process that depends strongly on the right hemisphere’s superior capacity for visuo-spatial transformations (Corballis, 1997).

The presence of Map 7 suggests a distinct cognitive component, since it occurred significantly later than Map 6, and the interaction between stimulus type and angular disparity had a different effect on its presence. While Map 6 was particularly sensitive to the presence of rotated hands, the cognitive processes associated with Map 7 are more difficult to discern. On the one hand, this map started earlier for rotated chairs than all other conditions. On the other hand, several other aspects such as duration, offset and AUC differentiated this map between upright and rotated stimuli. This map also had distinct current sources, with right temporal activations for rotated chairs, and left temporal activations for all other conditions. Though it is difficult to identify a specific cognitive process, the temporal activations and time-course of Map 7 suggest that this stage might be relevant for semantic classification of rotated and upright stimuli. In accordance with this, Schendan and Maher (2009) showed that after early and rapid visuo-perceptual processing of the stimulus, visual and semantic encoding continues and eventually temporally overlaps with later-occurring ERP patterns associated with MR.

Map 8 indicates the involvement of a third cognitive sub-process, which is mainly present for rotated conditions, irrespective of stimulus type. Source localization analyses revealed principally right prefrontal activations, reaching from the right frontal pole medially toward the anterior cingulate gyrus. This anterior localization, together with the relatively late occurrence of Map 8, is compatible with decision processes related to response selection (Seepanomwan et al., 2015). This interpretation is supported by previous studies which have similarly identified late electrophysiological components occurring after the spatial transformations during MR (Overney et al., 2005; Kong et al., 2018).

The main question motivating this study was whether MR of objects is grounded within motor emulation processes that mimic actual actions. An answer to this question rests on two main assumptions: first, that MR of hands activates motor processes similar to those activated during real hand actions; second, that MR of objects is sufficiently similar to MR of hands to trigger these same motor processes. Regarding the first assumption, functional imaging studies provide overwhelming evidence for overlapping activations between performed actions, imagined actions and MR of body parts (Richter et al., 2000; Wraga et al., 2005; Zacks, 2008; Milivojevic et al., 2009; Hardwick et al., 2018). Additionally, there is direct behavioral evidence for overlapping cognitive processes activated during real action and MR. For example, motor activity and MR produce almost indistinguishable patterns when motor rotation and MR are directly compared (Gardony et al., 2014), and even interfere with each other when they are aimed in opposite directions (Wexler et al., 1998; Wohlschlager and Wohlschlager, 1998; Wohlschlager, 2001). These findings strongly support the conclusion that MR not only “mimics” real manual rotation, but truly shares cortical resources as well as underlying cognitive components.

Regarding the second assumption, our electrophysiological results show that MR of objects and MR of hands are executed in a similar sequence of processing steps. Even though the temporal segmentation of EEG patterns suggests that Map 6 was particularly present for rotated hands, this is merely a relative difference and does not exclude presence of the processing steps associated with this map in the other conditions. Indeed, Map 6 is particularly interesting with respect to the possible involvement of motor processes because source analyses of this map not only revealed activations of the right angular gyrus, but also of dorsal primary motor and somatosensory cortex (see Figure 5). These EEG sources were present for both types of stimuli, and may be interpreted as an automatic activation of motor representations during the spatial transformation of mental images. Our findings thus not only complement previous fMRI studies by showing that brain regions necessary for motor execution are recruited during MR, but also show that this activation occurs simultaneously with the right angular gyrus, a brain region that is critical for spatial cognition. Of note, the recruitment of primary motor cortex (M1) at the stage of Map 6 (roughly at 290–360 ms) corresponds to the estimated involvement of M1 in hand rotation based on measures of cortical excitability, supporting a causal role of M1 in MR (Perruchoud et al., 2018).

In sum, our study reveals largely overlapping processing stages and sources of EEG activity when participants perform MR of objects or hands. In agreement with previous studies, we identified an early processing stage differentiating between the two stimulus types, which is compatible with a rapid stimulus classification mechanism. We additionally describe three later processing stages reflecting the spatial transformation associated with MR as well as decision processes related to response programming. Finally, our data show that one of these later stages generates activations of the right angular gyrus and primary motor/somatosensory cortex. These findings indicate that MR of objects and body parts recruit bodily representations and thus entails an emulation of low-level motor processes.

Though our study agrees with the findings of several previous EEG studies of MR, it also has some limitations that limit the scope of our conclusions. First, we used a very selective set of stimuli (hands and chairs) that may generate a specific pattern of electrophysiological responses. MR of other body parts (e.g., feet) or even the whole body, as well as other objects (e.g., manipulable items such as tools) might result in slightly different results. For example, it would be interesting to compare MR of hands to MR of feet, since both body parts have non-overlapping neural representations. A second question concerns the influence of gender. While we did not observe significant differences between female and male participants, this might be due to relatively small samples, since such differences have been found in previous studies. Finally, while recent studies have highlighted the influence of handedness in MR of hands (Cheng et al., 2020; Jones et al., 2021) our sample was restricted to right-handers. So far, our knowledge regarding the effect of handedness on neural processes involved in MR is insufficient. Finally, we tested performance with a task that essentially relies on egocentric transformations, limiting the extrapolation of the results to other tasks (e.g., tasks that afford object-centered transformations).

## Data availability statement

The datasets presented in this study can be found in online repositories. The names of the repository/repositories and accession number(s) can be found below: https://doi.org/10.26037/yareta:uoksutig4zaghb5i3s26dv5iqa and https://doi.org/10.26037/yareta:qkhx74c27vbmbh7qfbfog4jq4y.

## Ethics statement

The studies involving human participants were reviewed and approved by Commission Centrale d’Éthique du Canton de Genève. The patients/participants provided their written informed consent to participate in this study.

## Author contributions

MM conceived the design, performed the study, analyzed the results, and wrote the main manuscript text. AD supervised the study. GI analyzed the results. RP conceived the design and wrote the main manuscript text. All authors reviewed, corrected the manuscript, and approved the submitted version.

## Footnotes

1
https://sites.google.com/site/cartoolcommunity/home

2
http://doi.org/10.5281/zenodo.1164038
